# Human Papillomavirus E7 and p16^INK4a^ mRNA Multiplexed Quantification by a QuantiGene^TM^ Proof-of-Concept Assay Sensitively Detects Infection and Cervical Dysplasia Severity

**DOI:** 10.3390/diagnostics13061135

**Published:** 2023-03-16

**Authors:** Anna Sophie Skof, Lina Rotenberg, Paul Viktor Felix Hannemann, Sarah Thies, Eleonora Boschetti-Grützmacher, Andreas M. Kaufmann

**Affiliations:** Department of Gynecology, CVK, HPV Research Laboratory, Charité–Universitätsmedizin Berlin, Augustenburgerplatz 1, 13353 Berlin, Germany

**Keywords:** HPV test, Luminex suspension bead, cervical cancer screening, cervical cancer triage, molecular in vitro diagnostic

## Abstract

Background: Persistent infection with human papillomavirus (HPV) can lead to cervical cancer (CxCa). During the progression to CxCa, the expression of HPV oncogenes E6 and E7 is upregulated. In turn, cellular proteins such as p16^INK4a^ are also modulated. The combined detection of HPV oncogenes and cellular biomarkers indicative for dysplasia could be informative and convey better specificity than the current HPV tests that cannot discriminate transient infection from dysplastic changes. Methods: The QuantiGene^TM^ 2.0 Plex Assay platform was chosen for the effective multiplexing and quantitative detection of seven HPV-E7 mRNA targets (HPV6, 16, 18, 31, 45, 59, and 68) and the cellular mRNA of p16^INK4a^ as a biomarker for HPV-induced transformation. Actin-beta (ACTB) and hypoxanthine-guanine phosphoribosyltransferase 1 (HPRT1) were included as reference markers. Sequences for the specific capture and detector probes were customized and developed by ThermoFisher and formulated as a QuantiGene proof-of-concept (QG-POC) plex-set. The crude lysates of the HPV-positive cervical cancer cell lines CaSki (HPV16), HeLa (HPV18), MRHI-215 (HPV45), Erin59 (HPV59), ME180 (HPV68), and the HPV-negative cell line C33A, as well as liquid-based cytology smear samples (*n* = 441) were analyzed. The study was a proof-of-concept evaluating the feasibility of the platform. Logistic regression and receiver operating characteristic (ROC) analyses were performed to test for the sensitivity and specificity of HPV detection and dysplastic stage discrimination. Results: A QG-POC assay specifically and sensitively detects the HPV-E7 mRNA of seven different genotypes with an assay linearity between 20 and 13,000 cells. Cellular mRNA was detected from the crude lysates of cell lines and of cellular material from clinical liquid-based cytology smear samples. By combining HPV-E7 and p16^INK4a^ expression normalized to ACTB, high-grade dysplasia (HCIN) and invasive cervical cancer (CxCa) were detectable, discriminable, and correlated to the biomarker expression strength. The ROC analysis from the multivariate logistic regression model including HPV-E7 and p16 ^INK4a^ resulted in an AUC of 0.74, at the optimal cut-off (sensitivity: 70.4%; specificity: 66.0%) for HCIN detection. CxCa was detected with an AUC of 0.77 (sensitivity: 81.8%, specificity: 77.4%). Conclusions: The QG-POC assay is sufficiently sensitive to detect and quantify HPV-E7 and cellular mRNA species. Multiplexing allows the specific detection of at least 10 analytes in a single reaction. Determining the abundance of E7 and p16^INK4a^ transcripts when normalized to ACTB is informative about the presence of cervical dysplasia and potentially discriminates between low-grade and high-grade dysplasia and invasive cervical cancer. Further studies including more HPV genotypes and biomarkers are warranted.

## 1. Introduction

Cervical cancer (CxCa) is one of the most common female cancers, with an incidence of 600,000 new cases per year worldwide. The highest burden of disease is carried by low-and-middle-income countries (LMIC) where CxCa is frequently the leading cause of cancer deaths among women [1]. CxCa is generally caused by a human papillomavirus (HPV) infection [2]. Around 40 different HPV genotypes infect the anogenital epithelia [3]. They can be divided into low-risk HPV (lrHPV), which can cause benign condylomata, and high-risk HPV (hrHPV), which can cause invasive cancer. The World Health Organization (WHO) defines 12 HPV genotypes as hrHPV (i.e., HPV16, 18, 31, 33, 35, 39, 45, 51, 52, 56, 58, and 59). The genotypes HPV66, 68a, and 68b are defined as probable hrHPV and 26, 53, 73, and 82 as potential hrHPV [4].

The implementation of organized screening programs based on cytology testing has reduced the incidence and mortality of cervical cancer drastically [5]. However, cytology misses a lot of cases due to the low sensitivity of around 50% for detecting CIN2+. Several studies have shown that primary screening for HPV infections by PCR is more sensitive and cost-efficient than cytological-based screening [6,7,8,9]. For the development of a CIN lesion or CxCa it is necessary that an HPV infection persists. Most HPV infections are transient and clear within 12 months [10]. Since molecular HPV tests cannot discriminate between transient and persistent infections, the specificity for the detection of CIN2+ is low, leading to more referrals to colposcopy and follow-up [6]. Therefore, HPV primary screening is combined with cytology co- or reflex testing and additional triage tests are performed to categorize women according to their need for colposcopy and therapy. In personalized medicine, biomarker detection is an important tool for the risk stratification of patients and the diagnosis of pre-cancerous lesions and tumors [11]. In addition, in cervical (pre)-cancer screening and triage, biomarker detection assays are used to increase specificity and to identify those women who need further follow-up [12].

The progression to CxCa proceeds via various pre-malignant stages, histologically classified as cervical intraepithelial neoplasia (CIN) grades 1, 2, and 3. Cytological classification discriminates these stages as low- or high-grade squamous intraepithelial lesions (LSIL or HSIL) [13]. During transformation and progression, the expression of the viral oncogenes E6 and E7 is upregulated, as well as specific cellular biomarkers [14,15]. While low-grade lesions (CIN 1 and 2) show lower expression levels of the HPV oncogenes E6 and E7, in high-grade lesions and CxCa the transcripts are significantly more abundant [15]. The viral oncogenes E6 and E7 interact with the cellular protein p53 and the retinoblastoma protein (pRB), which leads to the degradation of p53 and the phosphorylation of pRB [16,17]. The phosphorylation of pRB leads to a release of the transcription factor E2F, which results in a compensatory overexpression of p16^INK4a^ [18]. This p16^INK4a^ overexpression correlates with hrHPV infection and with dysplasia severity which has been shown for mRNA and protein levels [14,15]. Moreover, low-grade CIN with no or only sparse p16^INK4a^ expression rarely progresses [19]. The quantification of biomarker expression, such as the HPV oncogenes E7 and E6, as well as p16^INK4a^, may therefore be promising for the detection of high-grade lesions and the progressive potential of low-grade dysplasia. It was shown for example, that the detection of the p16^INK4a^ protein with an ELISA has a high sensitivity for detecting CxCa [20]. The increasing expression level per cell of E6 and E7 with the severity of the neoplasia makes targeting the mRNA of the HPV oncogenes attractive to distinguish low- from high-grade dysplasia. The quantification of E6/E7 expression could be a way of identifying the severity of dysplasia at the molecular level, further improving the specificity of the assay.

Here, we report the utility of the QuantiGene^TM^ 2.0 Plex Assay platform technology for the multiplexed detection of HPV-E7 and cellular mRNAs from cultured cervical carcinoma cell lines and clinical smear specimens in a proof-of-concept study. The method uses the Luminex suspension bead array platform for read-out and branched DNA (bDNA) technology for signal amplification [21,22]. The aim of this study was to investigate the preliminary specificity and sensitivity of this innovative methodology and to determine whether the quantification of mRNA expression can provide information about the severity of dysplasia.

## 2. Materials and Methods

### 2.1. Cell Culture

Established HPV-positive CxCa cell lines were used to test the QuantiGene^TM^ 2.0 Plex Assay (ThermoFisher Scientific, Waltham, MA, USA) for the specific detection and sensitivity of the mRNA targets HPV-E7 and p16^INK4a^. The cellular mRNAs actin-beta (ACTB) and hypoxanthine phosphoribosyltransferase 1 (HPTR1) were used for normalization and quality control purposes. The cell lines CaSki (HPV16), HeLa (HPV18), ME 180 (HPV68), and C33A (HPV-negative) were obtained from American Type Culture Collection Inc. (Manassas, VA, USA) and CERV-215 (HPV45) was obtained from Cell Line Service (Eppelheim, Germany). The cell line Erin59 (HPV59) was kindly provided by Dr. D. Brown (University of Michigan Medical School, Indiana, IN, USA) [23]. The cell lines were cultured according to the distributor’s protocols. The cell culture media were supplemented with 10% fetal bovine serum (Biochrom AG, Berlin, Germany) and 5% penicillin-streptomycin (Biochrom AG, Berlin, Germany). The cells were cultured at 37 °C in a humidified 5% CO_2_ atmosphere until 70–80% confluence. The cell numbers were determined using a Countess automatic cell counter (Invitrogen GmbH, Carlsbad, CA, USA).

### 2.2. Clinical Study Samples

Liquid-based cytology smear samples were collected into a PreservCyt^®^ solution (Hologic, Marlborough, MA, USA) during the PIPAVIR study. Patients aged 30–60 years were recruited at an outpatient colposcopy clinic. Signed informed consent and ethical approval were obtained from the patients and the Bioethics Committee of the Medical School of Aristotle University of Thessaloniki/Greece (33–31/8/2012) and from the Ethikkommission der CHARITE-Universitätsmedizin Berlin/Germany (EA1/168/13), respectively. For each patient, an ectocervical and endocervical smear sample was collected using a CervexBrush^®^ (Rovers Medical Devices, B.V. Oss, The Netherlands) and a CytoBrush^®^ according to the manufacturer’s instructions, respectively. Cells were immediately fixed in ThinPrep containers (Hologic) containing 20 mL of PreservCyt^®^ solution. The cytology evaluation was performed by a central study cytopathologist [24]. The results were classified according to the Bethesda 2001 directive [13]. Women with positive results from cytology or the hrHPV test (Multiplexed Genotyping Assay) were referred to colposcopy. Biopsies for histological examination were only taken in case of abnormal findings, leading to only a small number of histological classified samples. Therefore, the results were graded using the ASSIST coding to summarize and weigh the cytological, colposcopic, and histological findings into four categories: normal (0), low-grade cervical intraepithelial neoplasia (LCIN) (1), high-grade cervical intraepithelial neoplasia (HCIN) (2), and CxCa (3) [25]. A subset of samples (*n* = 441) was selected from the main study, representing the different dysplasia stages, and including HPV16, 18, 31, 45, 59, 68, and HPV6 positively tested samples plus HPV-negative samples.

### 2.3. HPV DNA Testing

HPV genotyping was performed by Multiplexed Genotyping Assay (MPG assay) targeting the L1 gene sequence using a broad-spectrum GP5+/GP6+-bio primer set [26,27]. The MPG assay discriminates 18 hrHPV genotypes (HPV16, 18, 26, 31, 33, 35, 39, 45, 51, 52, 53, 56, 58, 59, 66, 68, 73, 82) and 9 lrHPV genotypes (HPV6, 11, 42, 43, 54, 57, 70, 72, 90). DNA was extracted using the DNA extraction mini-Kit (Qiagen, Hilden, Germany) with a final elution volume of 160 µL. Of this, 5 µL was used for the MPG assay PCR. The PCR products were hybridized to Luminex suspension beads conjugated with genotype-specific probes and stained with phycoerythrin-conjugated streptavidin (SAPE) as a reporter fluorochrome. The measurement was performed on a BioPlex 200 reader (BioRad, Hercules, CA, USA) and the result was given in median fluorescence intensity (MFI). A sample was considered positive if the MFI was three-fold higher than the median background of all the samples.

### 2.4. QuantiGene^TM^ 2.0 Plex Assay Design and Method Adaptation for QG-POC Assay

For mRNA quantification using the QuantiGene^TM^ 2.0 Plex Assay (ThermoFisher, Waltham, MA, USA), a custom plex-set was designed named QuantiGene proof-of-concept (QG-POC) targeting the following gene transcripts: HPV6-E7, HPV16-E7, HPV18-E7, HPV31-E7, HPV45-E7, HPV59-E7, HPV68-E7, and the generically upregulated cellular biomarker p16^INK4a^ (Table 1). ACTB and HPRT1 were included as reference gene transcripts. The HPV genotypes were chosen according to the availability of the corresponding cell lines.

**Figure 1:**
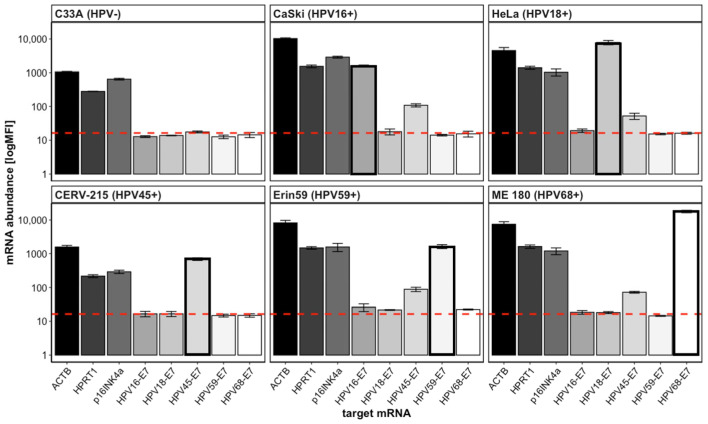
HPV-E7 and cellular mRNA detection and quantitation by QG-POC assay. Cell lysates representing approximately 4000 cells of the indicated cell lines were subjected to a QG-POC assay. Mean MFI (+/−SD, *n* = 3) values of the mRNA abundance are shown for the included target mRNAs. Bold framed columns indicate the E7 mRNA result for the intrinsic genotype of the given cell line. The dashed red line indicates the mean LOD (NC plus 3 × SD) of all the markers tested.

**Figure 2:**
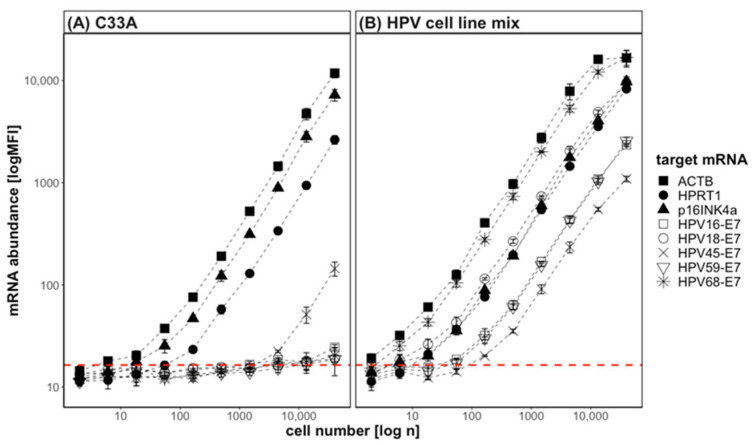
Limiting dilution titration of cell lines tested by QG-POC assay. The cell lysates of 40,000 cells per cell line were serially diluted 1:3 and subjected to a QG-POC assay. (**A**) Cell line C33A (HPV-negative). (**B**) HPV cell line mix containing 2 × 10^6^ cells/mL of each HPV-positive cell line (CaSki, HeLa, Erin59, Me180, CERV-215). MFI (+/−SD, *n* = 2) is shown. The dashed red line indicates the mean LOD of all the targets.

A crude cell lysate was used as assay input sample, which obviates the mRNA isolation steps. For this lysate, the cell line suspensions were counted, pelleted, resuspended at 1 × 10^6^ cells/mL in a working lysis buffer (1:3 diluted stock, Thermo Fisher Scientific, Waltham, MA, USA), and incubated at room temperature (RT) for 30 min. From the clinical Thinprep samples, 2 mL were centrifuged, the supernatant discarded, and the pellet lysed in 100 µL of the working lysis buffer for 30 min at RT (Supplemental Data Figure S1). For the QG-POC assay, 40 µL of the clinical sample lysates were used while the cell-line lysates were diluted as needed for certain cell numbers. Subsequently, 10 µL of the working bead-mix including the probe set, capture beads, blocking reagent, and proteinase K, according to the assay protocol, were added to the sample, mixed, and incubated at 54 °C and 600 rpm for 18–22 h (Supplemental Data Figure S1). During this step the target transcripts hybridized to specific DNA capture probes that were conjugated to color-coded Luminex beads. The next day, a signal amplification by a series of hybridization reactions of the pre-amplifier, amplifier, and label probe followed. SAPE was used to stain the biotinylated label probes. The fluorescence signals associated with individual capture beads and with specifically bound RNA probes were measured by a BioPlex 200 Luminex reader (BioRad) (Supplemental Data Figure S1). Signals were reported as MFI units and were proportional to the number of target RNA molecules bound to the capture beads. In every measurement, three negative controls (NC) were included. HPV positivity was defined by an MFI value above the limit of detection (LOD) that is the mean of the three NC plus three times their standard deviation (SD). For the easier visualization of HPV positivity in Figure 1 and Figure 2, the mean LOD from all the included markers was calculated. Due to many false-positive samples evaluated from the clinical smear samples, an empirical cut-off optimization was performed for the HPV genotypes 6, 18, 31, and 68, resulting in a new cut-off which was LOD plus 10 MFI for these genotypes. For the quantification analysis, the MFI of individual markers was corrected for the background by subtraction of the mean NC signal. The corrected MFI values were normalized to ACTB-expression MFI signals. Validation showed that an ACTB value of at least 100 MFI was needed for reliable normalization. The measurement of samples with a lower ACTB was repeated. The results are presented as relative median fluorescence intensity (rMFI).

### 2.5. Statistical Analysis

All statistical analyses were performed using R software [28]. For cleaning the data and graphic display, the R packages dplyr [29] and ggplot2 [30] were used. For the correlation analyses, Spearman’s rho (r_s_) was applied. The effect sizes were rated according to Cohen (1992) [31]. For comparison with HPV detection by the MPG assay, the concordance as well as Cohen’s kappa were calculated using the fmsb package [32]. The relative strength of agreement was described according Landis and Koch 2012 [33]. The univariate and multivariate regression analyses were performed using the glm()-function of the basic R software. Odds ratios (OR) were calculated to measure the association between the biomarker expression levels and the dysplasia stage. Receiver operating characteristic (ROC) analyses were performed using the pRoc software packages [34] for the individual analysis of the markers and ROCR [35] for the analysis of the multivariate logistic regression model. The patients’ dysplasia severity was classified using the ASSIST score [25]. Accordingly, the clinical stages tested were HCIN+ and CxCa [25]. For the correlation, concordance, ROC, and regression analyses, in multiple infections the “leading HPV” genotype was defined as the HPV genotype with the highest MFI value measured by the QG-POC assay.

## 3. Results

### 3.1. QG-POC Assay Detects mRNA Targets Specifically with Good Sensitivity in Cell-Line Samples

To verify the specificity of the designed QG plex-set for the nominal mRNAs and to preclude any cross-reactions between the HPV genotypes and/or cellular RNAs, we tested the lysates generated from 4000 cells of HPV-positive cervical cancer-derived cell lines CaSki (HPV16), HeLa (HPV18), CERV-215 (HPV45), Erin (HPV59), ME 180 (HPV68), and, as a specificity control, C33A (HPV-negative). While the expression over background was detected for the cellular mRNAs of ACTB, HPRT1, and p16^INK4a^ in all the cell lines investigated, the genotype-specific HPV-E7 signals appeared positive in the respective cell lines (Figure 1). Generally, these signals were specific and only detected in the HPV genotype-positive cell line and not in the HPV-negative C33A cells. Only the probe set for HPV45-E7 showed some cross-reactivity and a notable unspecific signal in all the cell lines that were positive for another HPV genotype, and also in C33A cells. For each HPV-positive cell line the correct respective HPV genotype was detected with a signal intensity significantly above the LOD (Figure 1, bold framed bar). The expression strength of the mRNAs varied between the different tested cell lines. For ACTB, the expression strength was between 1063 MFI in C33A and 10,312 MFI in CaSki. For HPRT1 and p16^INK4a^, the expression strength was the lowest in CERV-215, with 218 MFI and 293 MFI, respectively. The highest expression strength for HPRT1 was detected in ME 180 with 1634 MFI, and for p16^INK4a^ in CaSki with 2881 MFI. In addition, the expression levels of the respective target E7 mRNA varied between the cell lines. The lowest expression strength for the E7 mRNA was detected in CERV-215 for HPV45-E7 (693 MFI), and the highest in ME 180 for HPV68-E7 with 17,774 MFI. ME 180 also had the highest E7 mRNA strength when normalized to ACTB, which was regarded a marker for cellularity. The equivalent number of cells analyzed resulted in quite different MFI values in the different cell lines, both with and without normalization to ACTB. For example, the expression strength of the HPV-E7 mRNA detected in CaSki is one order of magnitude lower than in HeLa. This suggests that different target mRNAs may have different copy numbers in the cell lines investigated, which has been described before in cervical cancer cell lines [36].

After having established that the target mRNA sequences were specifically detected, and sufficiently strong signals of >1 order of magnitude above the LOD were detected when 4000 cells were tested, the assay sensitivity was further explored. A limiting dilution experiment was conducted by titrating the lysates of a cell-line mix (2 × 10^6^ cells/mL per HPV-positive cell line) and of the HPV-negative cell line C33A. The HPV–cell-line mix and the C33A-cell lysates were serially diluted at a ratio of 1:3 starting from 40,000 cells and subjected to a QG-POC assay (Figure 2). In C33A, the cellular mRNAs ACTB, HPRT1, and p16^INK4a^ were readily detectable as of approximately 6, 150, and 20 cells, respectively. An assay linearity of a three-fold MFI signal increase was given between this lower limit of detection and the highest values at 40,000 cells for all three markers. The background signals were consistent regardless of the number of cells in the assay. In the HPV–cell-line mix, the MFI signals for the E7 mRNA targets were specifically detected and increased linearly between 20 and 13,000 cells. In this range the accuracy of the three-fold change was between 80 and 120%. Therefore, quantifiable signals were given for all markers over 3–4 orders of magnitude of cell concentrations. Of note, the MFI signals obtained for ACTB, HPRT1, and p16^INK4a^ in the HPV–cell-line mix originated cumulatively from the five-fold cell number. For ACTB, a plateau was reached at the second dilution with a total cell amount of approx. 66,000 cells and an MFI value of around 25,000 MFI, while at the third dilution step (approx. 22,000 cells) the signal linearity was still given. This illustrates the wide range of cell concentrations that can be analyzed by this assay platform. For HPV45-E7 the assay linearity was given between 400 and 13,000 cells in the HPV–cell-line mix. This probe set for HPV45-E7 had shown an unspecific signal before and this again was seen in the C33A cells. As can be seen in Figure 2A, the unspecific signal is two orders of magnitude (three dilution steps) lower than the specific one in CERV-215 cells. Weak unspecific signals were detectable for the other E7 probe sets only at the highest concentration tested above 10,000 cells. In terms of assay sensitivity, only six cells were sufficient to detect HPV18-E7 and HPV68-E7 above the LOD from the HPV–cell-line mix. For HPV16-E7 and HPV59-E7, a signal above the LOD was achieved by approximately 55 cells, and for HPV45-E7 by 160 cells. Importantly, the MFI signals for the reference mRNA ACTB and HPRT1, the biomarker p16^INK4a^, and all HPV-E7 mRNAs were increasing with the same slope with increasing cell numbers. This allows the normalization of the signals within the linear range of each target mRNA to the cellular reference gene, and thus to the cellularity of the sample.

### 3.2. QG-POC Assay Detects HPV-E7 mRNA in Clinical Cervical Smear Samples

After having established the general functionality of the QG-POC assay on cell-line material and showing its remarkable sensitivity to detect as few as 10–50 cell equivalents, the next question was whether the clinical material from cervical smears could also be suitable for HPV detection. Clinical smear samples may contain mucus, blood, and other contaminants such as bacteria, yeast, or protozoa from the cervicovaginal microenvironment that could potentially interfere with the assay, thus an examination was crucial. Clinical cervical smear samples from 441 patients with a median age of 34.34 (18–74) years fixed in PerservCyt (Thinprep) were tested in this proof-of-concept study. The patients included in the diagnostic evaluation of the QG-POC assay were clinically classified using the ASSIST score as described by Agorastos et al. [25], which compiles and weighs the results from cytology, colposcopy, and histology for dysplasia grading. All the samples had been HPV-genotyped by MPG assay. We wanted to know if the cervical smear matrix in the crude lysate allows for sample use in the QG assay system, and if the E7 mRNA detection of the HPV genotypes represented in the QG-POC assay was possible.

Of the 441 patients included, 400 had tested positive by MPG for at least one of the following genotypes: HPV 6, 16, 18, 31, 45, 59, and 68. Additional HPV genotypes may have been present but were not regarded in this analysis. Importantly, we also wanted to investigate if the designed probe sets were functional and specific for the two genotypes hrHPV31 and lrHPV6, for which no cell lines were available. Therefore, clinical samples which tested positive for HPV6 or HPV31 by the MPG assay were also included. Due to the unspecific cross-reactivity of the HPV45-E7 probe set, HPV45-E7 was excluded from further analyses. A total of 41 women tested HPV-negative for any HPV infection. Out of the 400 HPV-positively tested patient smears, 223 had multiple infections, with 500 HPV infections for the included HPV genotypes in total. HPV16 was the most common type (73.0%), followed by HPV59 (12.0%) (Table 2).

The QG-POC assay includes probes for the E7-mRNA of six different hrHPV and HPV6 as an example of an lrHPV genotype. Compared with the highly target-amplifying PCR-based MPG assay, the signal-amplifying QG-POC assay detected less HPV infections cumulatively for the six HPV genotypes the assay was designed for, and more samples were HPV-negative (41 and 172, respectively). The proportion of HPV-negative tested samples in the QG-POC assay decreased with a higher dysplasia severity for the normal clinical groups, LCIN, HCIN and CxCa, of 60.0%, 36.2%, 22.6%, and 9.1%, respectively (Table 3). Conversely, this shows that the more severe the dysplasia, the higher the oncogene E7 expression, resulting in a higher percentage of positive samples by QG-POC assay. From the samples which tested negative in the MPG assay, the majority also tested negative in the QG-POC assay (95.1%, *n* = 39/41). In total, the QG-POC assay found 348 HPV infections in 269 patients. A total of 57 patients tested positive for multiple HPV infections, with two samples testing positive for all six of the included HPV genotypes. The most frequently detected genotype by the QG-POC assay was HPV16 in 44.0% of the samples (*n* = 194), followed by HPV59 (12.2%; Table 3). The samples that tested positive for HPV16 by QG-POC assay also tested positive for HPV16 by MPG assay, and 60.2% were positive in both assays. The other genotypes showed comparable positivity rates. For the CxCa samples in particular, and in contrast to the MPG assay, three samples were additionally HPV59-E7-positive, and one sample was HPV68-E7-positive. Three of these infections were detected with a very low MFI value and appear to be cross reactions with the leading HPV16-E7 infection detected. However, one HPV59-E7-positive CxCa sample had a very strong signal of 10,440 MFI. This sample was not detected by the MPG assay. Furthermore, two HPV16-positive CxCa samples were not detected by the QG-POC assay (Table 3).

Despite the low sample number for some HPV genotypes, we performed a correlation calculation since this was an exploratory study to obtain an impression of the QG-POC assay’s performance. Overall, positivity for the HPV genotypes included was lower in the non-target-amplifying probe-based QG-MPH assay than in the highly sensitive PCR target-amplifying MPG assay. The agreement between the MPG assay and QG-POC assay had a concordance of 70.29% for the six included genotypes (HPV6, 16, 18, 31, 59, 68) omitting the HPV45 data due to the unspecific probe set cross-reactivity. The correlation by Cohen’s kappa was 0.27 (CI 95% 0.17–0.38, *p* < 0.001) which represents a fair agreement (Table 4). A better agreement was found for individual genotypes, with the best agreement for HPV6-E7, with a concordance of 98.41% and a substantial agreement with a Cohen’s kappa of 0.66 (CI 95% 0.44–0.87, *p* < 0.001). Assay concordance for HPV18-E7 had the best Cohen’s kappa with 0.70 (CI 95% 0.58–0.83, *p* < 0.001), again a substantial agreement. Although HPV68-E7 had a high concordance between assays with 95.92%, no agreement was found with a Cohen’s kappa of −0.02, meaning the same samples were not detected positive by MPG assay and QG-POC assay for this HPV genotype. This was identified as a technical problem due to the probe design for the MPG and QG-POC assays (see discussion). The lowest concordance of 65.53% was found for HPV16-E7, with, however, a fair agreement (Κ= 0.35 (CI95% 0.26–0.43), *p* < 0.001). Since the QG-POC assay is a probe-based assay with no target amplification and only approximately 400-fold signal amplification, it was clearly less sensitive than the MPG assay, which is a PCR-based target amplification endpoint assay. Most clinical samples included in this study were positive for HPV16 by MPG assay and were associated with different ASSIST scores. Of note, non-detected HPV16-E7 by QG-POC assay was predominantly in the clinical group classified as “normal” with an ASSIST score of 0, or LSIL with an ASSIST score of 1, at 59.3% and 43.75%, respectively, and for both groups taken together as ≤LCIN, at 54.6%. For the ASSIST scores of 2 and 3, non-detected HPV16-E7 was 25.2% and 20%, respectively, and taken together as ≥HCIN, HPV16-E7 was negative only in 24.8%. This might correlate with the differential oncogene expression strength in dysplasia of progressive severity.

### 3.3. QG-POC Assay-Reported mRNA Expression Strength Indicates Dysplasia Presence and Severity

Having demonstrated that HPV-E7 mRNA detection of HPV genotypes is generally possible using the QG-POC assay from a cervical smear sample, we further explored the characteristics of such assay positivity. It was interesting to observe that the assay positivity correlated with dysplasia severity as more samples were detected positive when associated with a higher ASSIST score. Therefore, we wanted to investigate whether the detection of the mRNA-expression strength of the HPV-E7 oncogene, together with the biomarker p16^INK4a^ was indicative of the presence and severity of a dysplasia. According to the ASSIST scores, a total of 177 patients were within the normal limits (ASSIST score 0), 58 had a low-grade cervical intraepithelial neoplasia (LCIN, ASSIST score 1), 195 had high-grade cervical intraepithelial neoplasia (HCIN, ASSIST score 2) and 11 patients were diagnosed with invasive CxCa (ASSIST score 3) (Table 2).

**Figure 3:**
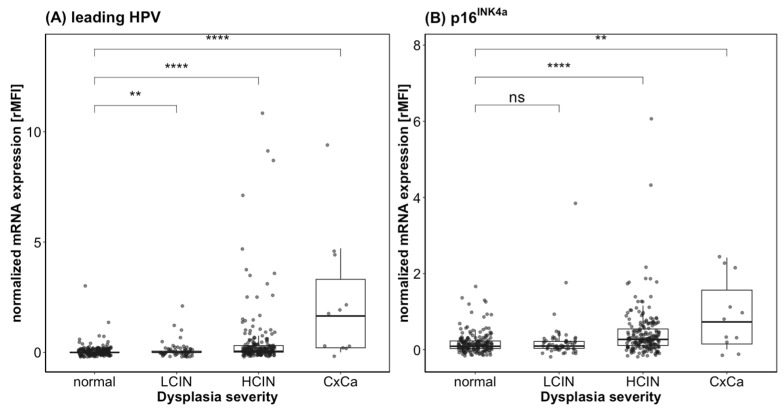
Biomarker-expression strength detected by QG-POC assay. mRNA expression of E7 of the leading HPV genotype (**A**) and p16^INK4a^ (**B**) for the clinical stages as classified for their severity by the ASSIST score. The relative expression of HPV-E7 (for the leading HPV type) and for p16^INK4a^ is shown as box whisker plots and dot plots for individual samples (gray dots) by the different dysplasia score categories. The MFI values are normalized to the reference marker ACTB resulting in relative MFI (rMFI). The *p*-values of the Wilcoxon test are shown for the group comparisons: ns, not significant, ** *p*< 0.01, **** *p* < 0.0001.

A papillomavirus infection can be caused by several genotypes simultaneously. It has been shown that each HPV genotype is linked to a discrete dysplasia and generally two viruses never infect the very same cell [37]. Such different lesions coexist, and it must be decided which is at the highest progression stage to evaluate the severity and determine by which HPV genotype it was induced. In the case of multiple HPV infections, it can be hypothesized that the HPV genotype with the strongest oncogene expression is responsible for the dysplasia with highest grade. Therefore, the genotype with the highest MFI signal for E7, i.e., the strongest expression, was defined as the “leading HPV”. This “leading HPV” genotype’s E7 expression and the expression of p16^INK4a^ as biomarkers for hrHPV infection and transformation were correlated with the ASSIST scoring to investigate their information content for dysplasia grading. Spearman’s rho correlation was performed and showed that the expression of the “leading HPV” and of p16^INK4a^ correlated positively with the ASSIST scoring, with an r_s_ of 0.47 (*p* < 0.001, *n* = 441) and 0.34 (*p* < 0.001, *n* = 441), respectively, which is rated as a medium-strong effect [31] (Figure 3). The E7-expression levels of the leading HPV genotypes were significantly different between the dysplasia-severity groups. For p16^INK4a^, there was no difference between the normal and the LCIN group (ASSIST score 0 and 1), i.e., very low to undetectable expression. In the HCIN and invasive carcinoma groups (ASSIST score 2 and 3), however, expression is markedly increased in both markers. Statistically significant differences between the normal/LCIN versus HCIN/CxCa severity grades were seen (Figure 3).

**Figure 4:**
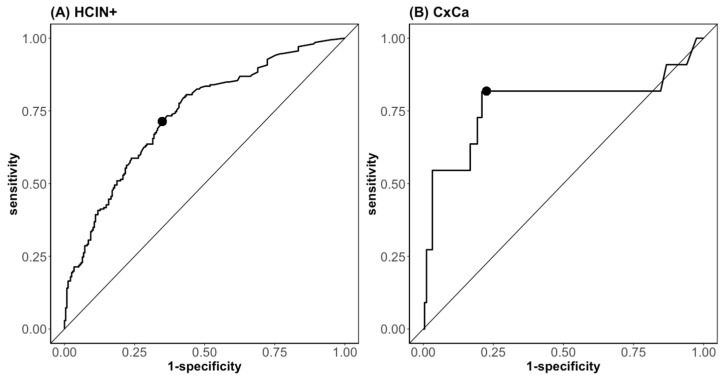
Accuracy of dysplasia detection by QG-POC assay. Shown are the ROC curves for the multivariate logistic regression models of the clinical borders (**A**) HCIN+ and (**B**) CxCa combining the expression strength of the biomarkers, leading HPV-E7 and p16^INK4a^. The points represent the best cut-off balance between sensitivity and specificity, i.e., accuracy. The diagonal represents the ROC of an uninformative marker or test.

A univariate logistic regression analysis was performed and showed an association for p16^INK4a^, HPV16-E7, and the leading HPV-E7-expression strength with the occurrence of high-grade lesions (HCIN+), with an OR of 5.81 (CI 95% 3.04–12.05, *p* < 0.001), 6.034 (CI 95% 2.55–18.37, *p* < 0.001), and 4.53 (CI 95% 2.44–9.78, *p* < 0.001), respectively. Because of the low numbers of patients testing positive for HPV68 and the potential probe problem, HPV68 was excluded from the regression analysis (Table 5). An ROC analysis for the individual markers showed that the most informative markers for distinguishing between HCIN+ and lower grades are p16^INK4a^, HPV16-E7, and the leading HPV-E7 with an AUC of 0.71 (CI 95% 0.66–0.76), 0.68 (CI 95% 0.63–0.72), and 0.74 (CI 95% 0.70–0.79), respectively. The sensitivity with the best cut-offs for these markers was 64.0%, 47.6%, and 63.6%, and the specificity was 68.9%, 84.7%, and 75.5%, respectively, in the sample sets investigated (Table 5). Because HPV16-E7 is integrated in the leading HPV-E7, the parameter p16^INK4a^ and the leading HPV-E7 were chosen for the multivariant logistic regression model. This combination detected HCIN+ with an AUC of 0.74, having a sensitivity of 70.4% and a specificity of 66.0% at the point of the best cut-off (Figure 4A).

An association of the expression strength of p16^INK4a^ or the leading HPV-E7 with the occurrence of CxCa had an OR of 2.08 (CI 95% 1.19–3.56, *p* < 0.01) and 1.51 (CI 95% 1.21–1.88, *p* < 0.001), and an AUC of 0.69 (CI 95% 0.48–0.91) and 0.79 (CI 95% 0.62–0.97), respectively (Table 6). These two parameters were included in the multivariate analysis. CxCa was detected by the biomarker combination of the leading HPV-E7 and p16^INK4a^ in the multivariate regression model with an AUC of 0.77, with a sensitivity of 81.8% and a specificity of 77.4% at the point of best cut-off (Figure 4B).

## 4. Discussion

The main objective of this study was to investigate as a proof of concept whether it is possible to detect and quantify the mRNA of seven HPV-E7 genotypes simultaneously in a multiplexed approach by using the QuantiGene^TM^ 2.0 Plex Assay technology. Experimental defined samples from cultured cervical carcinoma cell lines and authentic clinical cervical smear samples were analyzed. Interestingly, we found that by the quantification of the mRNA of HPV-E7 and the biomarker p16^INK4a^ information is provided about the presence and the severity of the underlying dysplasia.

The QG-POC assay was found to be a quick and easy method for the detection and quantification of mRNAs. The QuantiGene^TM^ 2.0 Plex Assay platform (ThermoFisher Scientific, Waltham, MA, USA) allows for the effective multiplexing of up to 80 targets with an ELISA-like workflow in a 96-well format, which reduces workload and costs. A crude lysate can be used as a sample, which obviates the isolation steps performed, for example, in PCR methods and thus makes the method less error and contamination prone [38]. The robust and low-complexity workflow together with the durable and relatively cheap reader device also allows for its use in places with limited resources. At the same time, the 96-well format, the workflow, and the use of magnetic beads allows for standardization and potentially automation. This distinguishes this assay platform from other mRNA-quantitating methods such as Nanostring, RNA seq, or RT-qPCR.

In this study we demonstrated that the QG-POC assay shows, in general, functionality on cultured cell line material. It detects the target mRNA sequences specifically with a sufficiently strong signal >1 order of magnitude above the LOD. Serial dilution experiments showed a very good assay sensitivity to detect as few as 10–50 cell equivalents. Assay linearity and therefore quantifiable results were given for all markers over three to four orders of magnitude of cell concentration, which allows for the measurement of samples with a wide range of cell concentrations. All the cell lines used in this study tested positive for p16^INK4a^, along with the hrHPV-positive and the hrHPV-negative cell line C33A as well. For the MFI signals for the reference mRNA, ACTB and HPRT1, the biomarker p16^INK4a^ and all HPV-E7 mRNAs increased with increasing cell numbers with the same slope. This is important because it allows for the normalization of the measured MFI values of the target mRNA to the reference markers ACTB and HPRT1, and thereby to the cellularity of the sample. This normalization results in a semi-quantitative relative MFI (rMFI) value which was used for further calculations. The probe set of HPV45-E7 showed unspecific positive signals in cell lines positive for another genotype and in high concentrations in the HPV-negative cell line C33A and was therefore excluded from further analyses. In subsequent studies the HPV45-E7 probe set needs to be redesigned and tested.

After establishing the assay principle with cultured cell lines, the study was extended to clinical smear samples. We found that HPV genotyping is generally possible from a crude lysate of clinical liquid-based cervical smear samples fixed in PreservCyt, and that the natural cervicovaginal matrix of these does not compromise the results. The QG-POC assay identified less HPV infections and more samples tested negative compared to the PCR-based MPG assay. This was expected since the MPG assay is a highly target-amplifying, endpoint PCR-based test that detects DNA, while the QG-POC assay is only signal-amplifying and detects mRNA. This means the method itself is less sensitive than the PCR and only detects HPV-E7 if the host cell is really infected and actively transcribes the oncogene to sufficient mRNA copy numbers. Therefore, the QG-POC assay evaluates more samples from the normal or HPV-negative LCIN sample groups than the MPG assay. However, the QG-POC assay also missed two HPV16-positive CxCa and 38 HPV16-positive HCIN samples, where the mRNA copy numbers should be high. Interestingly, the samples detected as HPV16-E7-negative by QG-POC also showed a significantly lower expression of p16^INK4a^ (data not shown), which potentially indicates a lower true dysplasia severity. The dysplasia severity could be overestimated by cytology and colposcopy as the histology results were missing in this study for many of the patients. Furthermore, only about 30–50% of the HSIL samples (CIN3) will eventually progress towards invasive CxCa over up to 30 years. The other half persists or regresses [39]. HPV16-E7 and p16^INK4a^ expression may better illustrate the biology of the lesion than viral DNA presence or morphology-based diagnostic methods and potentially may show a prognosis for the development of the respective lesion.

In total, a fair agreement between the MPG and QG-POC assays was found, with an overall concordance of 70.29% for the six included and analyzed genotypes (HPV6, 16, 18, 31, 59, 68). Potentially, further improving the sample lysis conditions could lower the background signals and increase the assay sensitivity in clinical smear samples, and this should be optimized especially for HPV16-E7 detection. In addition to the lower HPV-positivity, Cohen’s kappa showed no agreement for HPV68-E7 between the MPG and QG-POC assays. Two genetical subtypes exist, termed HPV68a and HPV68b [40]. It has been shown that different commercial tests detect different subtypes of HPV68 and not all identify both of them [41]. The MPG assay has a genotyping probe for HPV68a, while the QG-POC assay has a probe set that detects the HPV68b variant. Therefore, different positive results in the MPG and QG-POC assays for different samples could be a result of the differential detection of the two HPV68 subtypes. Further studies with more HPV68-positive samples should be performed to evaluate the HPV68-E7 probe set.

The quantification of the target mRNA is a means of evaluating whether the transcription and higher expression of the viral oncogenes corresponds to transformation events. We observed a significantly increased expression of HPV-E7 and of p16^INK4a^ in HCIN and CxCa samples compared to samples classified as normal without lesions and LCIN. This association has been described in studies before and has led to the hypothesis that testing the amount of mRNA is indictive of the presence of dysplasia and its severity [14,15,42]. Upon analysis of the biomarker expression strength and dysplasia severity, an association between p16^INK4a^, HPV16-E7, and the leading HPV-E7 with high-grade lesions (HCIN+) and remarkable ORs between 4.5 and 6 was found. Furthermore, the ROC analysis showed that these biomarkers have the potential to discriminate between high-grade dysplasia and low-grade dysplasia, with AUC values of 0.71 for p16^INK4a^, 0.68 for HPV16-E7, and 0.74 for the leading HPV-E7. Combining the biomarkers p16^INK4a^ and the leading HPV-E7 in a multivariate logistic regression model allowed a discrimination of the clinical stages for the border ≤LCIN and HCIN with an AUC value of 0.74 (sensitivity: 70.4%; specificity: 66.0%). For the discrimination of ≤HCIN and CxCa, the association between the expression strength of p16^INK4a^ and the leading HPV-E7 had an OR of 2.08 and 1.51, and resulted in an AUC of 0.69 and 0.79, respectively. The combination of these markers into a multivariate regression model increased the detection of CxCa with an AUC of 0.77, and with a sensitivity of 81.8% and a specificity of 77.4% at the point of best cut-off.

This proof-of-concept study used a restricted number of HPV genotypes in combination with only one cellular biomarker. Combining the leading HPV-E7 and p16^INK4a^ in a multivariant regression model resulted in a higher sensitivity (70.4%) compared to the sensitivity for the biomarkers alone (63.6% and 64.0%, respectively). However, specificity and assay accuracy did not benefit from this combination. The expression of p16^INK4a^ is directly affected by the expression of HPV-E7, so both markers are not independent parameters, and therefore the combination of these biomarkers is not necessarily advantageous [18]. A further aspect to consider with p16^INK4a^ is that its expression increases in an age-dependent manner, regardless of the presence or severity of dysplasia [43]. Furthermore, in our study cohort a weak but significant correlation (r_s_ = 0.15, *p* = 0.014) of p16^INK4a^ expression with the age of the patients was observed for all samples. This could be problematic for a universal cut-off development, as a woman at a higher age could have a false-positive test result, which would need to be validated. This naturally increased p16^INK4a^ expression, however, was less intense than that induced by hrHPV infection (Supplemental Data Figure S2). The addition of further biomarkers in the multivariate model could balance these problems and stabilize the model. Along with to p16^INK4a^, other cellular biomarkers such as Ki67, MCM2, Topo2α, and hTERT have been described as potential markers for cervical dysplasia detection, triage, and diagnosis [44,45]. Furthermore, there are more hrHPV genotypes that are responsible for CxCa development and could therefore be informative for dysplasia detection and characterization [4]. The use of the QuantiGene^TM^ 2.0 Plex Assay platform enables the multiplexed evaluation of up to 80 targets simultaneously in one single well. Therefore, the inclusion of additional hrHPV genotypes and virus-independent cellular biomarkers is possible to find the best biomarker combination for dysplasia detection and severity grading.

Both commercial DNA and mRNA-detecting HPV tests, have a higher sensitivity for detecting CIN2+ and CIN3+, but display lower specificity than this experimental QG-POC assay [46,47]. These commercial tests are PCR-based (DNA) and transcription-mediated amplification (TMA) mRNA-detecting endpoint tests that are highly target-amplifying and therefore not quantifying. Furthermore, they only detect HPV-derived nucleic acids and no additional cellular markers, except as internal controls. Moreover, the main limitation of this study was that only some of the included patients had a histological result, the others were classified with cytology or a combination of cytology and colposcopy using the ASSIST score to combine, unify, and weigh their clinical data [25]. Cytology has shown lower sensitivity, and colposcopy has also been shown to have lower specificity than histology [8,48]. Therefore, there are compromises in the evaluation of the sensitivity and specificity of the QG-POC assay in conjunction with the ASSIST score versus using the gold-standard histology result. A thorough comparison of the results presented from QG-POC with the comparator MPG-assay, clinical screening tests, cytology, and colposcopy will need samples from a larger study population with a higher number of HPV-negative patients. In addition, few HPV-negative samples for the specificity calculation and only a small number of CxCa cases could be investigated in this study, limiting the significance of the results for CxCa detection. Nevertheless, our results show a high potential to improve the specificity of HPV screening tests by combining the mRNA quantification of HPV-E7 and cellular biomarkers. Furthermore, the results indicate that the mRNA-expression strength is not only indicative of the presence of an active HPV infection, but also the severity of the dysplasia. Further studies are warranted to better define the sensitivity and specificity of the QG-POC assay, also with an extended HPV-genotype panel.

## 5. Conclusions

In this proof-of-concept study we demonstrated the high potential of the QG-POC assay to detect cervical dysplasia and simultaneously indicate its severity in one step from a liquid-based cytology cervical smear sample. Further studies are warranted to investigate if the integration of other biomarkers further improves the assay specificity and diagnostic classification. Additionally, a study with more and better characterized samples is needed to develop risk scores and validate the best marker combination.

## Figures and Tables

**Table 1:** Target mRNAs and gene accession numbers for the custom design of the QG-POC assay plex-set.

Target mRNA	Gene Accession Number
HPV6-E7	L41216
HPV16-E7	K02718
HPV18-E7	X05015
HPV31-E7	J04353
HPV45-E7	X74479
HPV59-E7	EU918767
HPV68-E7	Y14591
ACTB	NM_001101
HPRT1	NM_000077
p16^INK4a^	NM_000194

**Table 2:** Characterization of HPV infections in clinical samples by MPG assay.

ASSIST Score ^1^	Clinical Group	HPV Genotype ^2^ (*n* (%))						No HPV Detectable
		16	18	31	59	68	6	
0	Normal (*n* = 177)	113 (52.6)	15 (6.9)	6 (2.8)	26 (12.1)	2 (0.9)	5 (2.3)	41 (23.2)
1	LCIN (*n* = 58)	48 (63.2)	6 (7.9)	8 (10.5)	5 (8.6)	4 (5.3)	2 (2.6)	0 (0)
2	HCIN (*n* = 195)	151 (62.7)	25 (10.4)	24 (10.0)	22 (9.1)	4 (1.7)	5 (2.1)	0 (0)
3	CxCa (*n* = 11)	10 (90.9)	1 (9.1)	0 (0)	0 (0)	0 (0)	0 (0)	0 (0)
total	441	322 (73.0)	47 (10.7)	38 (8.6)	53 (12.0)	8 (1.8)	12 (2.7)	41 (9.1)

^1^ ASSIST score: composite result score of the patients’ cytology, colposcopy, and histology findings. ^2^ HPV genotypes included in the QG-POC assay. HPV, human papillomavirus; LCIN, low-grade intraepithelial lesion; HCIN, high-grade intraepithelial lesion; CxCa, invasive cervical cancer; MPG, multiplexed genotyping.

**Table 3:** HPV infections detected in clinical samples by QG-POC assay.

ASSIST Score ^1^	Clinical Group	HPV Genotype * (*n* (%))						No HPV Detectable
		16	18	31	59	68	6	
0	Normal (*n* = 177)	46 (26.0)	8 (4.5)	11 (6.2)	16 (9.0)	3 (1.7)	5 (2.8)	106 (60.0)
1	LCIN (*n* = 58)	27 (46.6)	4 (6.9)	5 (8.6)	12 (20.7)	2 (3.4)	3 (5.1)	21 (36.2)
2	HCIN (*n* = 195)	113 (57.9)	20 (10.3)	26 (13.3)	23 (11.8)	4 (2.1)	6 (3.1)	44 (22.6)
3	CxCa (*n* = 11)	8 (72.7)	2 (18.2)	0 (0.0)	3 (27.3)	1 (9.1)	0 (0.0)	1 (9.1)
total	441	194 (44.0)	34 (7.8)	42 (9.5)	54 (12.2)	10 (2.3)	14 (3.2)	172 (39.0)

* HPV45-E7 excluded due to unspecific signals; ^1^ ASSIST score: composite result score of the patients’ cytology, colposcopy, and histology findings; HPV, human papillomavirus; LCIN, low-grade intraepithelial lesion; HCIN, high-grade intraepithelial lesion; CxCa, invasive cervical cancer.

**Table 4:** Concordance between MPG and QG-POC assay for detected HPV genotypes.

		Cohen’s Kappa		
HPV Type	Concordance (%)	Κ	CI (95%)	Agreement ^+^
6	98.41	0.66 ***	0.44–0.87	Substantial
16	65.53	0.35 ***	0.26–0.43	Fair
18	95.46	0.73 ***	0.61–0.84	Substantial
31	93.42	0.60 ***	0.46–0.74	Moderate
59	79.37	0.03	−0.14–0.20	Slight
68	95.92	−0.02	−0.48–0.44	No
QG-POC HPV genotypes ^#^	70.29	0.27 ***	0.17–0.38	Fair

^#^ 6 HPV genotypes included in QG-POC assay: HPV6, 16, 18, 31, 59, 68; *** *p* < 0.001, HPV45 excluded due to unspecific signals by the HPV45-E7 probe set; ^+^ rating according to Landis and Koch 2012 [33].

**Table 5:** ROC analysis and univariate and multivariate logistic regression for high-grade dysplasia HCIN+.

Biomarker	AUC (CI 95%)	Cut-off	Sensitivity (%)	Specificity (%)	Univariate Regression OR (CI 95%)	Multivariate Regression OR (CI 95%)
HPV16-E7	0.68 (0.63–0.72)	0.025	47.6	84.7	6.03 *** (2.55–18.37)	-
HPV18-E7	0.54 (0.50–0.57)	0.015	16.9	90.2	3.13 (1.29–18.89)	-
HPV31-E7	0.53 (0.49–0.57)	0.005	17.5	88.9	1.30 (0.22–9.57)	-
HPV59-E7	0.50 (0.48–0.53)	0.005	1.9	100	3.88 (0.85–140.64)	-
Leading HPV-E7	0.74 (0.70–0.79)	0.490	63.6	75.5	4.53 *** (2.44–9.78)	2.78 ** (1.55–5.97)
p16^INK4a^	0.71 (0.66–0.76)	0.175	64.0	68.9	5.81 *** (3.04–12.05)	3.31 ** (1.64–7.13)

** *p* < 0.01, *** *p* < 0.001; HPV, human papillomavirus; HCIN+, high-grade intraepithelial lesion or higher; AUC, area under the curve; ROC, receiver operating characteristic; CI, confidence interval; OR, odds ratio.

**Table 6:** ROC analysis and univariate and multivariate logistic regression for CxCa.

Biomarker	AUC (CI 95%)	Cut-off	Sensitivity (%)	Specificity (%)	Univariate Regression OR (CI 95%)	Multivariate Regression OR (CI 95%)
Leading HPV-E7	0.79 (0.62–0.97)	0.185	81.8	78.6	1.51 *** (1.21–1.88)	1.49 * (1.06–2.01)
p16^INK4a^	0.69 (0.48–0.91)	0.725	54.5	90.2	2.08 ** (1.19–3.56)	1.06 (0.48–2.31)

* *p* < 0.05, ** *p* < 0.01, *** *p* < 0.001; HPV, human papillomavirus; CxCa, invasive cervical cancer; AUC, area under the curve; ROC, receiver operating characteristic; CI, confidence interval; OR, odds ratio.

## Data Availability

The data presented in this study are available on request from the corresponding author. The data are not publicly available due to ethical restrictions.

## Supplementary Materials

The following supporting information can be downloaded at: https://www.mdpi.com/article/10.3390/diagnostics13061135/s1, Figure S1: Schematic illustration of the QG-POC assay workflow; Figure S2: p16^INK4a^ expression strength detected by QG-POC assay in relation to age, clinical stage, and HPV positivity.
