# Supplementary material for: Human Papillomavirus E7 and p16INK4a mRNA Multiplexed Quantification by a QuantiGeneTM Proof-of-Concept Assay Sensitively Detects Infection and Cervical Dysplasia Severity
Source: Diagnostics (Basel). 2023 Mar 16;13(6):1135. doi: 10.3390/diagnostics13061135 (PMC10047034; doi:10.3390/diagnostics13061135)
Supplement: Supplementary file 1 [file diagnostics-13-01135-s001.zip › diagnostics-2222672-supplementary.pdf]

Supplementary Materials:

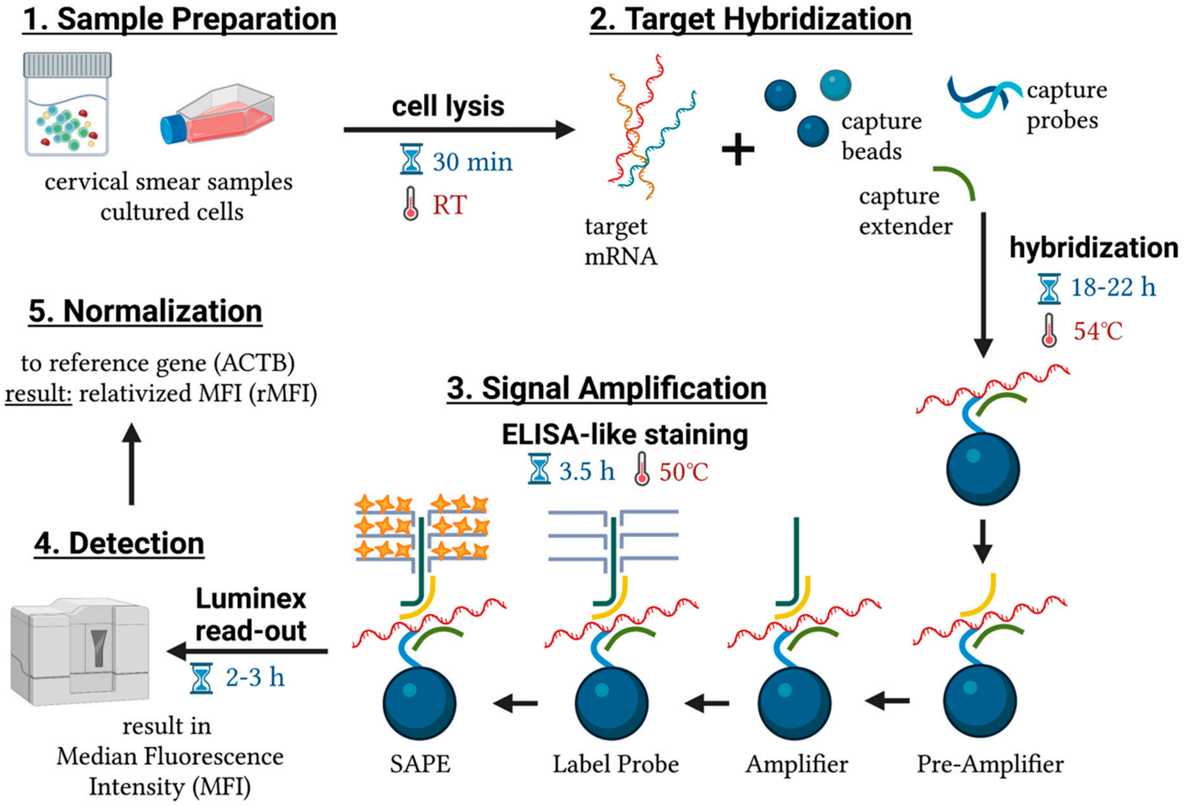

Figure S1. **Schematic illustration of the QG-POC assay workflow. 1. Sample Preparation: cultured cell lines and cervical smear samples were lysed, and crude lysates were used in setting up the assay. 2. Target Hybridization: during the incubation of the lysates with capture probes, capture beads and capture extenders, the targeted transcripts hybridize to the specific RNA capture probes that are conjugated to color-coded Luminex beads. 3. Signal Amplification: a series of hybridization reactions of Pre-amplifier, Amplifier, and Label Probe follows. Phycoerythrin-conjugated streptavidin (SAPE) is used as reporter binding to the biotinylated Label Probes. 4. Detection: the fluorescence signals associated with individual capture bead classes and proportional to the captured mRNA target is measured by a Luminex reader. Results are reported as Median Fluorescence Intensity (MFI). 5. Normalization: MFI results are normalized to ACTB expression signals as indicator of cellularity. Results are presented as relative Median Fluorescence Intensity (rMFI). (Created with BioRender.com)**

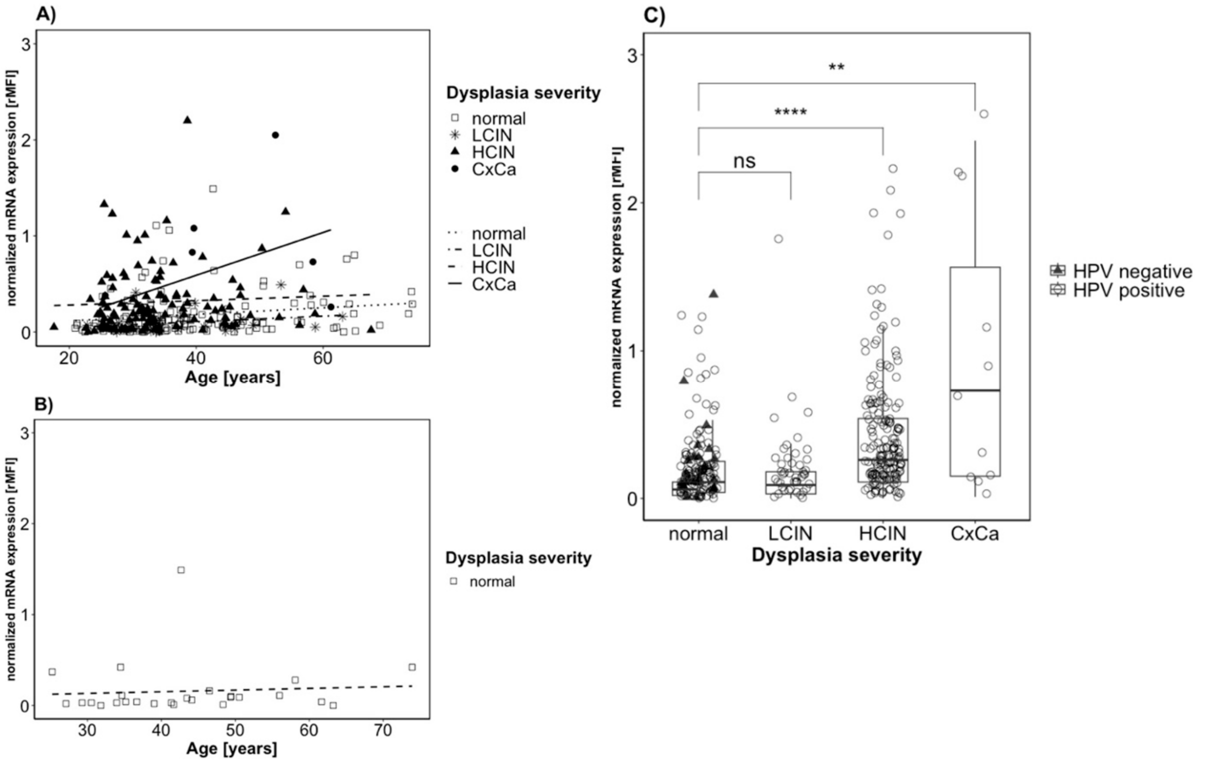

Figure S2. p16^{INK4a} expression strength detected by QG-POC assay in relation to age, clinical stage, and HPV positivity. p16^{INK4a} expression is upregulated with higher dysplasia severity, but also increases with higher age of the patients. This effect can be measured in HPV negative samples without dysplasia. To investigate whether the age dependency is present in our sample set and if it influences the use of p16^{INK4a} as a biomarker for dysplasia detection we plotted A) The normalized mRNA expression of p16^{INK4a} against the age in years of the patients by dysplasia severity classified by the ASSIST score. Regression lines are shown for the different dysplasia severity groups. B) The normalized mRNA expression of p16^{INK4a} against the age of the patients in years for the HPV negative samples without dysplasia. Hatched line, regression curve for normal, HPV negative patients. C) The relative expression of p16^{INK4a} is shown as box whisker plots and dot plots for individual samples by the different dysplasia score categories. Samples are also categorized by HPV positivity. MFI values are normalized to the reference marker ACTB resulting in relative MFI (rMFI). The y-axis was set to the same scale to allow direct comparison. P-values of Wilcoxon-test are shown for group comparisons, ns= not significant, ** p< 0.01, **** p<0.0001.
